# Egr-1 Upregulates Siva-1 Expression and Induces Cardiac Fibroblast Apoptosis

**DOI:** 10.3390/ijms15011538

**Published:** 2014-01-21

**Authors:** Karin Zins, Jiri Pomyje, Erhard Hofer, Dietmar Abraham, Trevor Lucas, Seyedhossein Aharinejad

**Affiliations:** 1 Laboratory for Molecular Cellular Biology, Center for Anatomy and Cell Biology, Medical University of Vienna, Vienna A-1090, Austria; E-Mails: karin.zins@meduniwien.ac.at (K.Z.); dietmar.abraham@meduniwien.ac.at (D.A.); seyedhossein.aharinejad@meduniwien.ac.at (S.A.); 2 Molecular Vascular Biology, Department of Vascular Biology and Thrombosis Research, Vienna Competence Center, Vienna Medical University, Vienna A-1090, Austria; E-Mails: j.pomyje@skc.de (J.P.); erhard.hofer@meduniwien.ac.at (E.H.)

**Keywords:** cardiac fibroblasts, apoptosis, gene expression, transcription factor, Egr-1, Siva-1

## Abstract

The early growth response transcription factor Egr-1 controls cell specific responses to proliferation, differentiation and apoptosis. Expression of Egr-1 and downstream transcription is closely controlled and cell specific upregulation induced by processes such as hypoxia and ischemia has been previously linked to multiple aspects of cardiovascular injury. In this study, we showed constitutive expression of Egr-1 in cultured human ventricular cardiac fibroblasts, used adenoviral mediated gene transfer to study the effects of continuous Egr-1 overexpression and studied downstream transcription by Western blotting, immunohistochemistry and siRNA transfection. Apoptosis was assessed by fluorescence microscopy and flow cytometry in the presence of caspase inhibitors. Overexpression of Egr-1 directly induced apoptosis associated with caspase activation in human cardiac fibroblast cultures *in vitro* assessed by fluorescence microscopy and flow cytometry. Apoptotic induction was associated with a caspase activation associated loss of mitochondrial membrane potential and transient downstream transcriptional up-regulation of the pro-apoptotic gene product Siva-1. Suppression of Siva-1 induction by siRNA partially reversed Egr-1 mediated loss of cell viability. These findings suggest a previously unknown role for Egr-1 and transcriptional regulation of Siva-1 in the control of cardiac accessory cell death.

## 1. Introduction

Cardiac fibroblasts are the predominant cell type (by number) in healthy heart tissue [1] secreting structural collagen, providing an extracellular matrix and supporting the function of contractile cardiomyocytes [2–4]. In addition to the role played by cardiomyocytes, fibroblasts are intrinsically involved in processes that lead to heart failure [5]. In cardiac pathology, fibroblasts play a key role in remodeling of the left ventricle which leads to dilatation and subsequent heart failure [6,7]. Hypertension induced ventricular hypertrophy is also associated with fibroblast hyperplasia [8] and apoptosis of cardiac fibroblasts has recently been identified as an essential process during drug induced ventricular remodelling [9]. Recently, a pivotal cardioprotective role has been demonstrated for fibroblasts in myocardial hypertrophy and the prevention of heart failure [10]. Cardiac fibroblasts proliferate, migrate and remodel the interstitium by modulating the secretion of extracellular matrix components and matrix metalloproteinases with multiple signaling cascades controlling ECM synthesis, ECM degradation, fibroblast proliferation and apoptosis [11]. In addition, recent data indicate that cardiac fibrosis is possibly regulated by inducing apoptosis in fibroblasts [12].

Apoptosis or programmed cell death is a fundamental process in tissue homeostasis. Cellular apoptosis may be induced by extrinsic and intrinsic factors that often culminate in the activation of a family of cysteine protease (caspase) zymogens that proteolytically degrade cellular substrates [13,14]. Induction and susceptibility to cellular apoptosis is tissue and cell type specific and dependent on the proteome which is primarily under the control of transcription factors [15]. In contrast to necrosis, apoptotic cells are rapidly recognized and eliminated by neighboring phagocytes to prevent inflammation [16]. Apoptosis is associated with the development of the cardiovascular system and cardiovascular disease [17] and is responsible not only for the removal of cardiomyocytes during heart failure [18] but also non-myocyte components [19] which are the predominant apoptotic cellular compartment in heart failure [20].

In this context, the early growth response gene Egr-1 [21] has been identified as an important mediator of fibroblast activation and pathology [22]. Egr-1 is a transcription factor containing zinc finger DNA-binding motifs and domains to both activate and repress transcription [23]. As master switches controlling developmental processes, transcription factors are attractive candidates as intrinsic regulators of cellular fate including cardiac cell reprogramming [24]. Also known as NGFI-A [21], ZIF268 [25], Tis8 [26] and Krox24 [27], Egr-1 cell specifically modulates phenotype through the regulation of over 300 target genes [28,29]. Induced variously by growth factors, hormones and during stress and inflammatory responses, Egr-1 mediated transcription brings together a multitude of signaling cascades vital for growth, differentiation and apoptosis [23]. Egr-1 mediates these transcriptional effects by binding to high affinity 5′-TGCGTG/AGGCGG/T-3′ [30] and GC-rich 5′-GCGG/TGGGCG-3′ [31,32] motifs in target gene promoters. Cellular Egr-1 activity is tightly controlled by the NGFI-A binding proteins (Nab-1 and Nab-2) which inhibit Egr-1 activity by binding of *N*-terminal Nab-conserved domains to the transcriptional repression domain of Egr-1 [33]. Although Nab-1 is expressed almost ubiquitously, Nab-2 is a delayed immediate response gene transcriptionally regulated by Egr-1 in a transcriptional feedback loop [34]. Expression of Egr-1 has been previously linked to several aspects of cardiovascular pathology including intimal thickening following acute vascular injury [35], cardiac hypertrophy [36], atherosclerosis [37] and angiogenesis [38]. Doxorubicin induced cardiomyopathy is also mediated by Egr-1 [39] and targeting Egr-1 reduces the pathological effects of acute myocardial infarction in rats [40]. The phenotypes associated with Egr-1 are cell-type specific [35] and increased Egr-1 expression has been linked to the induction of apoptosis [41].

Influencing cardiac fibroblast activity and viability therefore has enormous potential therapeutic benefit in a range of myocardial malignancies [2,42]. However, little is known about the role of increased Egr-1 expression on induction of apoptosis in cardiac fibroblasts, which could play a role in pathologic processes in cardiac tissue.

In the present study, we investigated the role of Egr-1 expression in cardiac fibroblasts. Over-expression of Egr-1 induced apoptosis sensitive to caspase activation involving dissipation of the mitochondrial membrane potential in primary human cardiac fibroblasts *in vitro.* Apoptosis was accompanied by transcriptional regulation of the co-repressor Nab-2. In a screen of pro-apoptotic genes that could potentially be activated by Egr-1 [28], we identified up-regulation of Siva-1 during the Egr-1 mediated apoptotic induction phase, a gene that has been associated with myocardial apoptosis [43]. Treatment with siRNA targeting Siva-1 partially reversed Egr-1 mediated cytotoxicity. These data suggest a previously unknown role for Egr-1 and Siva-1 in the control of interstitial cell apoptosis in cardiac tissue.

## 2. Results and Discussion

### 2.1. Results

#### 2.1.1. Egr-1 Induces Caspase Sensitive Cardiac Fibroblast Apoptosis *in Vitro*

**Figure 1.**
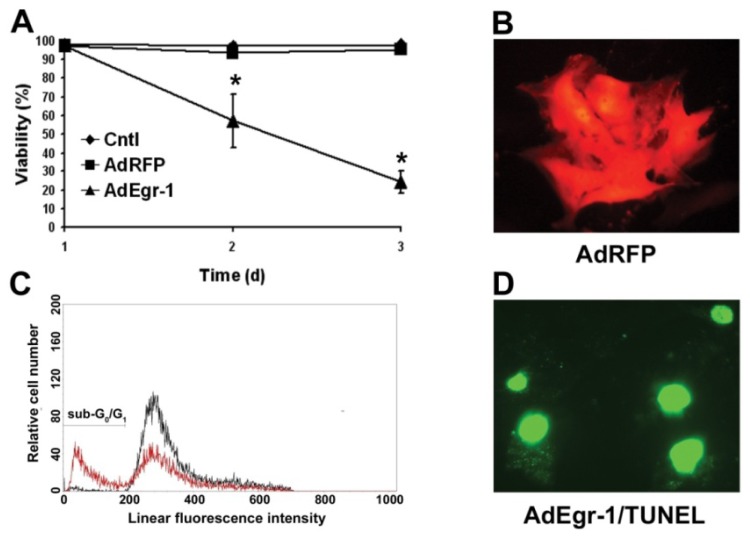
Egr-1 induces cardiac fibroblast apoptosis *in vitro*. (**A**) Cardiac cultures were infected with AdEgr-1 or AdRFP, washed after 24 h and viabilities assessed by trypan blue exclusion compared to untreated controls (Cntl). Data incorporate the standard deviation of three experiments (* *p* = 0.005 at day 2 and *p* < 0.0001 at day 3); (**B**) AdRFP (magnification ×40) infected control cultures; (**C**) Representative FACS cell cycle analysis showing the development of sub-G_0_/G_1_ apoptotic DNA content in cultures infected with AdEgr-1 (red histogram) compared to AdCntl (black) after 48 h; and (**D**) AdEgr-1 infection was associated with the induction of apoptosis demonstrated by TUNEL positive nuclei (magnification ×40). No staining was seen in control cultures (data not shown).

To examine the potential role of Egr-1 expression in cardiac accessory cells, human cardiac fibroblasts were isolated and infected with recombinant adenovirus (AdEgr-1), and compared to a reporter virus expressing RFP (AdRFP) or empty virus (Adcntl) control cultures. Infection of cardiac fibroblasts with AdEgr-1 resulted in the development of membrane compromised adherent cells 48 h post-infection (Figure 1A). In contrast, AdRFP infection was not toxic (Figure 1A) and RFP was robustly expressed by cardiac fibroblasts (Figure 1B). To determine the mechanism of cell death induced by Egr-1 over-expression, cells were isolated and cell cycle analysis was performed. As shown in Figure 1C, in Egr-1 infected cultures a sub G_0_/G_1_ phenotype develops 48 h post-infection characteristic of cellular apoptosis. Cells on chamber slides were then fixed 48 h post-infection and analyzed for DNA strand breaks by TUNEL staining. A typical result is shown in Figure 1D demonstrating incorporation of fluorescein dUTP by terminal deoxynucleotidyl transferase into free 3′-OH DNA moieties characteristically generated during the DNA fragmentation phase of apoptosis.

**Figure 2.**
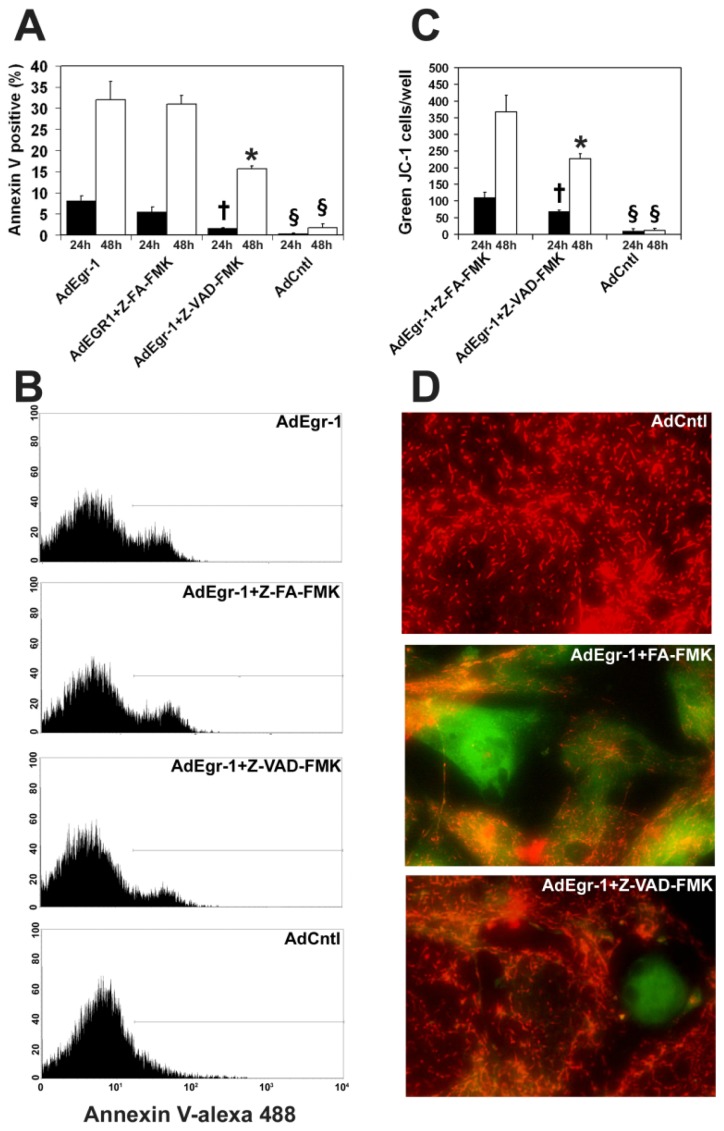
Apoptosis induced by Egr-1 in cardiac fibroblasts is caspase dependent. (**A**) AdEgr-1 induction of apoptosis 24 and 48 h post-infection assessed by binding of alexa 488 conjugated annexin V in FACS analyses is inhibited by the pan-caspase inhibitor Z-VAD-FMK when compared to the negative Z-FA-FMK control or AdEgr-1 cultures (**†** *p* < 0.005, * *p* < 0.0005). Empty adenovirus (AdCntl) cultures were significantly different from all AdEgr-1 cultures (§ *p* < 0.001). Experiments incorporate the standard deviation of three experiments; (**B**) Representative FACS histograms 24 h post-infection; (**C**) Staining with JC-1 shows AdEgr-1 induces mitochondrial membrane depolarization that is partially inhibited by addition of Z-VAD-FMK when compared to the negative Z-FA-FMK control (**†** *p* < 0.005, * *p* < 0.0005). Empty adenovirus (AdCntl) cultures were significantly different from all AdEgr-1 cultures (§ *p* < 0.001); and (**D**) Representative diagrams showing JC-1 staining (magnification ×60).

We then determined whether Egr-1 mediated apoptotic induction is sensitive to caspase inhibition. AdEgr-1 induced apoptosis, as assessed by binding of annexin V in fluorescence-activated cell sorting (FACS) analysis, was significantly inhibited, although not abrogated, by the pan-caspase inhibitor Z-VAD-FMK 24 (*p* < 0.005) and 48 h (*p* < 0.0005) post-infection when compared to the Z-FA-FMK control peptide and empty adenovirus control cultures (Figure 2A,B). In addition, quantification of apoptotic events in cell cycle analysis (Figure 1C) showed that AdEgr-1 treatment induced 32.1% ± 2.52% sub G_0_/G_1_ events in the cardiac fibroblast population. Comparable results were observed in the presence of the Z-FA-FMK control peptide (29.92% ± 4.5%) but were significantly reduced in comparison to both cultures in the presence of Z-VAD-FMK (14.9% ± 0.6%; *p* < 0.005) 48 h post-infection. In AdCntl infected cultures and all 24 h post-infection cultures, the G_0_/G_1_ population was always less than 2.4%. To assess whether Egr-1 involves the mitochondrial pathway of apoptosis, we assessed loss of mitochondrial transmembrane potential (ΔΨ_m_) in the presence of the cationic dye JC-1 which fluoresces as red aggregates in intact mitochondria and green as cytoplasmic monomers in cells undergoing apoptosis [44]. As shown in Figure 2C,D, AdEgr-1 infection induced loss of ΔΨ_m_ which was partly delayed 24 (*p* < 0.005) and 48 h (*p* < 0.0005) post-infection in the presence of Z-VAD-FMK when compared to the Z-FA-FMK control peptide. Together, these results show that sustained over-expression of Egr-1 induces cardiac fibroblast apoptosis which is partly reversed by caspase inactivation and involves loss of ΔΨ_m_.

#### 2.1.2. Egr-1 Mediated Transcriptional Regulation during Cardiac Fibroblast Apoptosis

**Figure 3.**
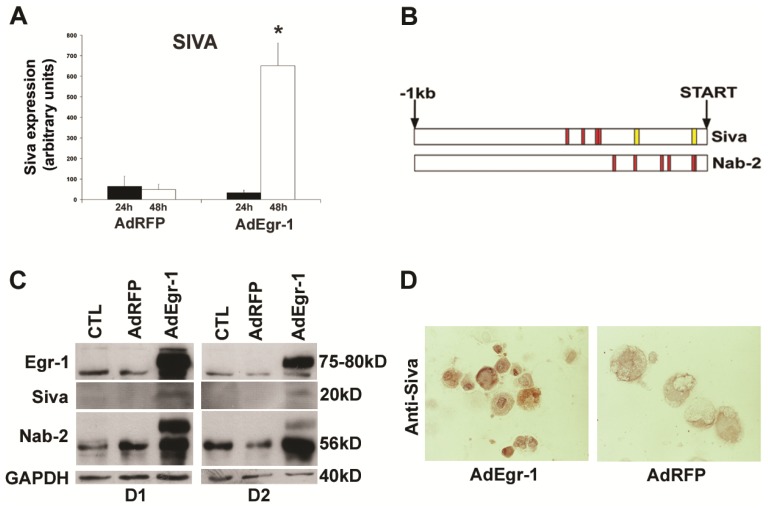
AdEgr-1 induces downstream target gene expression in cardiac fibroblasts. **(A**) In cardiac fibroblast cultures, monitoring mRNA levels of Siva-1 mRNA by real time RT-PCR 24 and 48 h post-infection with AdEgr-1 and AdRFP controls showed transient induction of Siva-1 mRNA after 48 h. Levels of all transcripts were normalized against B2M. Values incorporate the standard deviation of three experiments (* *p* < 0.005 compared to all other groups); (**B**) Bioinformatic analysis comparing the promoter regions of human Siva-1 and Nab-2 reveal the presence of multiple high affinity (yellow) and GC rich (red) Egr-1 binding sites relative to the transcriptional start site; (**C**) In cardiac fibroblast cultures, over-expression induced by AdEgr-1 infection is accompanied by expression induction of Nab-2 24 (D1) and 48 h (D2) post-infection and Siva-1 protein on D2 compared to AdRFP infected cultures and untreated controls as demonstrated by Western blotting; and (**D**) Induction of Siva-1 expression 48 h post-infection with AdEgr-1 *in vitro* compared to AdRFP infected cardiac fibroblast cultures stained with anti-Siva-1 antibody by immunohistochemistry (magnification ×40).

We then tested the hypothesis that Egr-1 may induce the transcription of a pro-apoptotic genetic program. To evaluate the transcriptional response associated with Egr-1 overexpression in cardiac fibroblasts, we next assessed the expression of potential pro-apoptotic downstream regulator molecules that could potentially play a role in Egr-1 mediated apoptosis. During induction of apoptosis by AdEgr-1 induced by a 26-fold increase in mRNA, we found that the transcriptional target gene Siva-1 undergoes a transient 10 ± 1.2 fold induction 48 h (*p* < 0.005) after infection with AdEgr-1 (Figure 3A).

The Siva-1 protein contains a domain homologous to a death domain and binds the cytoplasmic tail of ligand stimulated members of the TNF receptor superfamily including CD27 and the glucocorticoid induced TNF receptor [45]. Siva-1 can induce caspase-dependent apoptosis and is also localised to mitochondria and inhibits the anti-apoptotic activity of Bcl-X_L_ and Bcl-2 [46]. Siva-1 is known to be upregulated during apoptosis [47] and is a known transcriptional target of the tumor suppressor p53 [48] and the transcription cofactor TIP30 [49].

To investigate whether Siva-1 expression could be potentially influenced by Egr-1 through interaction with 5′ regulatory sequences, we then retrieved and analysed the putative Siva-1 promoter region and compared this to the Nab-2 corepressor which is strongly regulated by Egr-1 in a negative feedback loop [34]. Nab-2 is the major inducible transcriptional repressor of Egr-1 with the ability to inhibit Egr-1 transcriptional activity [33] via direct interaction with Egr-1 [50].

Transcriptional induction by Egr-1 is mediated by binding to high affinity 5′-TGCGTG/ AGGCGG/T-3′ [30] and GC-rich 5′-GCGG/TGGGCG-3′ [31,32] motifs in target gene promoters. We monitored Nab-2 expression as a secondary marker of Egr-1 regulation because in contrast to Nab-1, basal levels of Nab-2 are strongly under the control of Egr-1 [51]. Both promoters contain multiple binding sites for the GC rich recognition motif [31]. As shown diagrammatically in Figure 3B, the Nab-2 promoter contains GC motifs at base pairs 35–51, 41–57, 120–136, 147–163, 243–259 and 332–348 proximal to the transcriptional start site whereas the Siva-1 promoter has four consensus sequences at 370–386, 376–392, 428–444 and 477–493. The Siva-1 promoter also contains two additional high affinity consensus sequences [30] at 41–57 and 241–257 in the 5′ regulatory region. These data indicate that the Siva-1 promoter region contains elements that are known to be bound by Egr-1 to induce transcription.

We then assessed the protein expression of these molecules during the induction of apoptosis *in vitro*. Basal expression of both Egr-1 and Nab-2 was observed at the protein level in cardiac fibroblast cultures indicating that Egr-1/Nab-2 transcriptional pathways are normally active in these cells. In comparison to baseline expression levels in unstimulated cells, Egr-1 expression was increased 26-fold on the mRNA level (data not shown). Normal cardiac fibroblasts, however, lack expression of Siva-1 protein (Figure 3C). Sustained over-expression with AdEgr-1 induces expression of both the Nab-2 repressor and the Siva-1 protein during the apoptotic induction phase. Subsequent decreases in expression on day 3 for all molecules can be attributed to over 80% cell death in cultures at this time point (Figure 1A).

Cytoplasmic and nuclear induction of Siva-1 expression is also seen 24 h post-infection with Ad-Egr-1 in cells with condensed cytoplasm characteristic of apoptosis (Figure 3D). Taken together, these results show that sustained expression of Egr-1 elicits downstream transcriptional regulation of the Egr-1 repressor Nab-2 and the pro-apoptotic Siva-1 gene product during the induction phase of apoptosis in cardiac fibroblasts *in vitro*.

#### 2.1.3. Suppression of Siva-1 Expression Reduces Egr-1 Mediated Cardiac Fibroblast Cell Death

**Figure 4.**
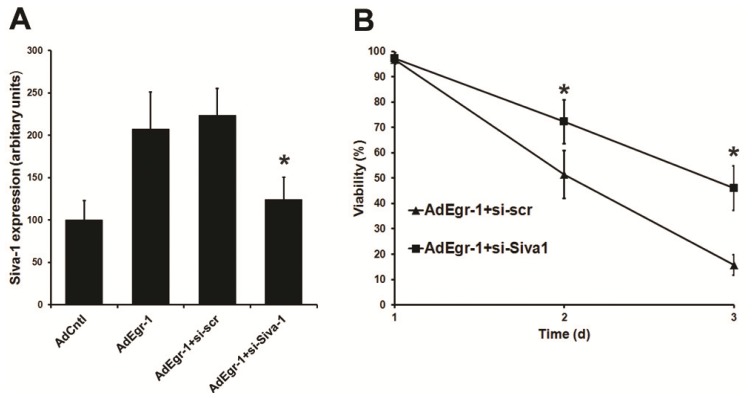
Suppression of Siva-1 expression reduces Egr-1 mediated cardiac fibroblast apoptosis. (**A**) Prior transfection with siRNA directed against Siva-1 (si-Siva-1) reduces Siva-1 mRNA 48 h post AdEgr-1 infection compared to scrambled siRNA controls (si-scr) as assessed by real time RT-PCR; and (**B**) Transfection with si-Siva-1 increases cell viability in AdEgr-1 infected cultures compared to si-scr controls. Data incorporate the standard deviation of three experiments (* *p* < 0.05).

To assess whether Siva-1 expression induction contributes to Egr-1 mediated induction of cell death, cardiac fibroblasts were transfected with siRNA targeting sequences in exon 4 of Siva-1 (si-Siva-1) prior to infection with AdEgr-1 and compared to controls treated with scrambled siRNA (si-scr). Transfection with si-Siva-1 significantly reduced the induction of Siva-1 mRNA expression 48 h after infection with AdEgr-1 by 89.2% compared to scrambled controls (Figure 4A). Since AdEgr-1 treated cardiac fibroblasts become membrane compromised rapidly following binding of annexin V (data not shown), we subsequently used dye exclusion to monitor apoptosis in cultures. Transfection with si-Siva-1 significantly (*p* < 0.05) retarded the loss of viability in AdEgr-1 cultures compared to si-scr controls (Figure 4B). These data indicate that Egr-1 mediated induction of cell death is influenced by expression of Siva-1.

### 2.2. Discussion

The transcription factor Egr-1 is involved in the regulation and expression of more than 300 genes [29] and acts as a trigger and a convergence point for many signaling cascades. The resulting pleiotropic effects manifest in seemingly divergent physiological processes, including cellular growth, differentiation, inflammation and apoptosis [35].

Egr-1 also plays a role in a variety of cardiovascular pathological processes such as intimal thickening following acute vascular injury [35] and it has been shown to regulate expression of molecules critically linked to cardiac hypertrophy [36], atherosclerosis [37] and angiogenesis [38]. In human endothelial cells, Egr-1 regulates the expression of a variety of genes, suggesting that Egr-1 is a key mediator of inflammation and apoptosis in vascular cells [29]. Elevated myocardial Egr-1 expression has been correlated to cardiac allograft rejection [52], coronary allograft vasculopathy [53] and Egr-1 induces myocardial ischemic/reperfusion injury [54]. A recent study investigating the role of Egr-1 in the pathogenesis of myocardial ischemia-reperfusion injury showed that Egr-1 silencing at the time of reperfusion following acute myocardial ischemia decreases myocardial inflammation and apoptosis leading to improved cardiac function [36]. A role for increased expression of Egr-1 and the Nab corepressors in pathogenic cardiology have also been identified [41,55]. Repression of Egr-1 transcription by increased pathological cardiac expression of Nab-1 inhibits cardiac growth and cardiomyocyte hypertrophy [56].

Doxorubicin induced cardiomyopathy is also mediated by Egr-1 down-regulation of the sarco (endo) plasmic reticulum calcium-ATPase [39] and DNAzymes targeted against Egr-1 reduce the pathological effects of acute myocardial infarction in rats [40]. Although the cellular transcriptional machinery to enable Egr-1 expression is differentially present in cardiac cells [57], the underlying pathology of increased Egr-1 expression is unlikely to be linked to contractile cells since transgenic animals overexpressing Egr-1 under the control of the differentiated cardiac myocyte-specific muscle creatine kinase promoter undergo normal cardiac development [58]. Egr-1 deficient mice also show no cardiac abnormalities [59] and cardiomyocyte Egr-1 expression has even a cardioprotective role against catecholamine infusion by regulation of the sodium-calcium exchanger-1 in these mice [58].

In close contact with contractile elements, interstitial cardiac fibroblasts are the most numerous cell type in the heart and play key roles in a variety of cardiac pathologies. The data presented here indicate that Egr-1 and the co-repressor Nab-2 are constitutively expressed in human cardiac fibroblasts and that sustained adenoviral-mediated overexpression of Egr-1 induces apoptosis independent of endogenous Nab-2 upregulation during the induction phase *in vitro*. These observations highlight a physiological function for Egr-1 signaling independent of the co-repressor Nab2 in regulating apoptosis.

The potential Egr-1 driven transcriptional regulation during the apoptotic induction phase in cardiac fibroblasts *in vitro* was then investigated by screening for expression of putative pro-apoptotic Egr-1 target genes. We found a strong transient upregulation of Siva-1 mRNA and protein that may be controlled by the high affinity and GC-rich binding sites for Egr-1 in the putative Siva 5′ regulatory region identified *in silico*. Siva can induce both intrinsic and receptor mediated extrinsic apoptosis [47].

The Siva protein contains a domain homologous to a death domain and induces apoptosis through interaction both with members of the tumor necrosis factor receptor superfamily [45] and anti-apoptotic members of the Bcl-2 family of proteins [60]. Siva also plays a role in apoptotic induction induced by oxidative stress [61] and is a transcriptional target of the tumor suppressor p53 [48]. The Siva protein is strongly induced during cardiac cell apoptosis after infection with the coxsackievirus B3 virus which may lead to heart failure [43]. Expression of Siva-1 protein has been described in the cytoplasm [60], to be partly localized to mitochondria [61] and is expressed in the nucleus [46]. Although Siva-1 was only partially responsible for the AdEgr-1 induced apoptosis in cardiac fibroblasts, no induction of potential Egr-1 pro-apoptotic target genes such as death effector domain-containing protein-2 or dual-specificity phosphatase [28] was found in AdEgr-1 infected cardiac fibroblasts illustrating the cell-specific regulatory mechanisms governing Egr-1 transcriptional control of cell death.

Taken together, these data suggest that increased Egr-1 expression could pathologically alter cardiac fibroblast homeostasis by downstream regulation of target genes. The Egr-1/Siva-1 signaling axis could therefore serve as targets to counteract apoptosis in cardiac tissue. Future studies in animal models are necessary to extend our understanding of the key pathophysiological roles of Egr-1/Siva-1 signaling in cardiac fibroblasts. This will help to identify pathologic cardiovascular conditions possibly regulated by Egr-1/Siva-1 in humans which could be targeted with Egr-1/Siva-1–specific strategies.

## 3. Experimental Section

### 3.1. Recombinant Adenoviral Constructs and Infection

The monomeric red fluorescent protein (RFP) DSred2 (BD Biosciences, Palo Alto, CA, USA) and the human Egr-1 coding sequence, including the single intron amplified from the PAC clone E13873Q3 (library number 704, RZPD, Berlin, Germany), were subcloned into pACCMVplpASR+ under the control of the immediate early CMV promoter. Empty vector and cloned products were then cotransfected with the replication deficient adenoviral pJM17 genome into trans-complementing HEK293 as described previously [62,63] and adenovirus (AdCntl, AdRFP and AdEgr-1) were purified by consecutive cesium chloride centrifugations and plaque formation estimated with agarose overlays.

### 3.2. Culture, Infection and Transfection of Cardiac Fibroblasts

Normal human ventricular cardiac fibroblasts were purchased from Lonza and cultivated in Fibroblast Growth Medium-3 (FGM-3; Lonza, Basel, Switzerland) at 37 °C in a fully humidified air atmosphere containing 5% CO_2_. Sub-confluent fibroblasts in 6-well plates and 8-well chamber slides (BD Biosciences, Palo Alto, CA, USA) were infected with adenovirus (AdCntl, AdRFP or AdEgr-1) for 24 h in culture medium, the medium was then changed and cells were analysed at the indicated time points post infection. Cells were transfected in serum free medium for 4 h in the presence of Lipofectamine 2000 (Life Technologies, Grand Island, NY, USA) with scrambled control (UCGUCAGGACGAGUGUCAU) and siRNA directed against Siva-1 exon 4 (UUCUCGUACAUGU CACUGC) obtained from Life Technologies. Cells were then infected with adenovirus as before. Commencing with viral infection, the caspase inhibitor Z-VAD-FMK and control peptide Z-FA-FMK (BD Biosciences, Palo Alto, CA, USA) were added (100 μM) throughout cultures where indicated.

### 3.3. Assays of Viability and Cell Death

Culture viabilities were estimated by nuclear staining in 0.1 μg/mL 4′-6-diamidino-2-phenylindole (DAPI) or propidium iodide *in situ* and by trypan blue exclusion in harvested cells. Cells were also sequentially incubated in binding buffer with biotin-conjugated annexin V (Bender Medical systems, Vienna, Austria) and alexa 488-conjugated streptavidin (Molecular Probes, Carlsbad, CA, USA) for 15 min at room temperature and then incubated in 2 μg/mL propidium iodide to discriminate between early and late apoptotic events. Loss of mitochondrial membrane potential (ΔΨ_m_) was analysed by fluorescence microscopy in triplicate subconfluent cultures in 8-well chamber slides. Cultures were incubated with the potentiometric dye JC-1 (5,5′,6,6′-tetrachloro-1,1′,3,3′-tetraethylbenzimidazolyl carbocyanine iodide) for 15 min at 37 °C according to the manufacturer’s instructions (Peninsular laboratories, San Carlos, CA, USA) and results expressed as green JC-1 cells per well.

For cell cycle analysis, cell pellets were fixed in 70% ethanol at 4 °C for 1 h, washed twice and resuspended in PBS supplemented with and 0.1% DNase-free RNase A and 100 μg/mL propidium iodide. Scatter gates were set to exclude sub-cellular particles and at least 10_4_ events analyzed on a FACScan flow cytometer (BD Biosciences, Palo Alto, CA, USA) with an argon laser tuned at 488 nm. Fluorescein based TUNEL assays were performed according to the manufacturer’s protocol (Roche, Basel, Switzerland). Briefly, adherent cells in chamber slides were washed in PBS, air dried and fixed with 4% paraformaldehyde in PBS (pH 7.4) for 1 h at room temperature, permeabilized for 2 min on ice with 0.1% Triton X-100 in 0.1% sodium citrate and incubated with terminal deoxynucleotidyl nucleotide transferase in preformed cacodylate buffer containing fluorescein dUTP for 1 h at 37 °C, rinsed with PBS and mounted in Cityfluor and analysed on a fluorescent microscope (Zeiss, Thornwood, New York, NY, USA).

### 3.4. Promoter Analysis

The 1 kb proximal to the reference sequence transcriptional start sites of human Siva-1 (U82938.1) and NAB-2 (NM_005967.2) were retrieved from GenBank genomic contigs and analyzed with MatInspector software [64] from Genomatix (Munich, Germany).

### 3.5. Real Time RT-PCR Analysis

Total RNA was isolated from cardiac fibroblast cultures in TRIZOL (Life Technologies, Grand Island, NY, USA), reverse transcribed from an oligo dT-primer with maloney murine leukemia virus reverse transcriptase (Thermo Scientific, Waltham, MA, USA) and PCR performed with FastStart DNA Master SYBR Green mix (Roche Diagnostics, Indianapolis, IN, USA) as described previously [65]. The temperature profile included initial denaturation (10 min at 95 °C), 45 cycles of denaturation (15 s at 95 °C), annealing (5 s at 58–62 °C) and elongation with fluorescence monitoring (16 s at 72 °C) with the Siva (5′-TTCAGAACCACACGGCTAC-3′/5′-TTCCTCTCTTTTTCCTCCC-3′) and B2-microglobulin (B2M) (5′-GATGAGTATGCCTGCCGTGTG-3′/5′-CAATCCAAATGCGGCATCT-3′) primer sequences. Analyses were performed on a Lightcycler (Roche, Mannheim, Germany, 2001) and data were analyzed with LCDA Version 3.5.3 (Roche). Specificity of amplification products was determined with melting curve analyses [66]. Standard curves for expression of each gene were generated by serial dilution of known quantities of respective cDNA gene templates. Gene expression levels were quantified by normalization to the B2M signal.

### 3.6. Western Blotting and Histochemistry

Western blotting of cell lysates (50 μg) was performed as described previously [67] with polyclonal rabbit anti Egr-1, rabbit anti Nab-2 and goat anti-Siva (Santa Cruz, Santa Cruz, CA, USA), horseradish peroxidase (HRP) conjugated secondary antibodies (GE Healthcare Life Sciences, Pittsburgh, PA, USA) and mouse monoclonal HRP conjugated anti-GAPDH antibody as loading control (Abcam, Cambridge, UK). For immunohistochemical staining, cytospins were air dried, fixed in acetone for 8 min at 4 °C, rehydrated in PBS 5% horse serum and sequentially incubated with goat anti-Siva (Santa Cruz, Santa Cruz, CA, USA), biotinylated horse anti-goat Ig (Vector, Burlingame, CA, USA) and HRP conjugated streptavidin (Dako, Glostrup, Denmark), developed with DAB chromogen (Vector), dehydrated and mounted in DPX.

### 3.7. Data Analysis

We used the Student’s *t* test and ANOVA to compare the data between the groups. All statistical tests were two-sided. Statistical tests were done with the use of SPSS software (version 20, SPSS Inc., Chicago, IL, USA, 2011). Data are expressed as means ± SD. *p* values < 0.05 were considered to indicate statistical significance.

## 4. Conclusions

In this study, we show that Egr-1 is constitutively expressed by cardiac fibroblasts. Sustained overexpression induces rapid induction of apoptosis associated with the activation of caspases and collapse of the mitochondrial membrane potential. Sustained overexpression of Egr-1 leads to induction of proapoptotic Siva-1 expression. Suppression of Siva-1 induction significantly retards loss of cell viability indicating Siva-1 in the induction of apoptosis process. Thus, Egr-1 mediated induction of Siva-1 provides a novel regulatory pathway affecting apoptosis in cardiac fibroblasts, which may be involved in regulation of cardiac pathology.
